# Complex Effects of Vitamin E and Vitamin C Supplementation on *in Vitro* Neonatal Mononuclear Cell Responses to Allergens

**DOI:** 10.3390/nu5093337

**Published:** 2013-08-26

**Authors:** Heather J. H. Wassall, Graham Devereux, Anthony Seaton, Robert N. Barker

**Affiliations:** 1 Division of Applied Medicine, University of Aberdeen, Aberdeen, AB25 2ZD, UK; E-Mail: r.n.barker@abdn.ac.uk; 2 Division of Applied Health Sciences, University of Aberdeen, Aberdeen, AB25 2ZD, UK; E-Mail: g.devereux@abdn.ac.uk; 3 Environmental and Occupational Medicine, University of Aberdeen, Aberdeen, AB25 2ZD, UK; E-Mail: a.seaton@abdn.ac.uk

**Keywords:** α-tocopherol, neonatal, allergens, T-cells

## Abstract

Low maternal dietary vitamin E (but not vitamin C) intake during pregnancy has been associated with increased *in vitro* cord blood mononuclear cell (CBMC) proliferative responses, childhood wheezing and asthma. We investigated whether these associations reflect direct effects of vitamin E by investigating the effects of supplementing CBMC cultures with physiological concentrations of vitamin E. CBMC from seventy neonates were cultured supplemented with either nothing, α-tocopherol or ascorbic acid. Proliferative, IFN-γ, IL-4, IL-10 and TGF-β responses were measured. In general, vitamin E supplementation was associated with a trend for reduced proliferative responses after stimulation with antigens and house dust mite, and with increased proliferation after stimulation with timothy grass allergen. There was a trend for CBMC cultures to exhibit decreased secretion of IFN-γ, IL-10 and IL-4. Supplementation with vitamin C had no effect on CBMC proliferation, but increased IFN-γ and IL-4 production, and decreased IL-10 production. In conclusion, *in vitro* vitamin E and C supplementation of CBMC modifies neonatal immune function, but not in a manner predicted by observational epidemiological studies. The observed associations between vitamin E and childhood respiratory disease are complex, and the nature and form of nutritional intervention need to be carefully considered before inclusion in trials.

## 1. Introduction

The prevalence of asthma and atopic disease has risen dramatically in recent decades and it has been hypothesised that declining dietary antioxidant intake has contributed to the increase [1,2]. *In vitro* data support this notion by demonstrating that antioxidant deficiency can promote T-helper (Th) cell differentiation towards the Th2 phenotype [3,4,5]. However, trials of antioxidant supplementation of adults with atopic disease suggest minimal clinical benefit [6]. This is not unexpected in the light of recognition that early life factors are important in the immunopathogenesis of atopic disease, with compartmentalisation of allergen specific Th cell immunity into adult equivalent Th1 and Th2 patterns occurring in most children before the age of 5 years [7]. Moreover for vitamin E Th-cells from younger subjects are more responsive to vitamin E and naïve (CD45RA(+)) Th-cells are more responsive to vitamin E than Th cells with a memory/activated phenotype (CD45RO(+)) [4,8].

Three birth cohort studies have reported reduced maternal dietary vitamin E intake during pregnancy to be associated with an increased likelihood of childhood wheezing [9,10] and asthma [11]. Moreover, two of these cohorts have demonstrated that low maternal vitamin E intake during pregnancy is associated with increased *in vitro* proliferative responses by cord blood mononuclear cells (CBMC) and that this association is independent of the potentially confounding effects of birth order, sex, maternal atopy and maternal smoking [12,13]. Based on these findings, there have been calls for trials of vitamin E based intervention during pregnancy [14]. Although keen to conduct such an intervention trial we considered it important to conduct preliminary work to justify/refute the use of vitamin E supplements for several reasons. Firstly, antioxidant supplement trials for many other diseases have produced negative or adverse results despite encouraging observational data [15]. These disparities between observational and intervention studies have been attributed to a failure to appreciate the complex differences between individuals with high and low antioxidant intakes [16]. Secondly, although CBMC responses are associated with maternal dietary vitamin E intake during pregnancy, there is no association with maternal or cord blood α-tocopherol [12]. These considerations raise the possibility that the observed epidemiological association between maternal vitamin E intake during pregnancy and CBMC responses may not be a direct association but merely a consequence of confounding by other nutrients associated with vitamin E.

To investigate these issues further, and to inform any intervention trial, we conducted an *ex vivo* study to test whether the observed epidemiological association between reduced maternal vitamin E intake during pregnancy and increased CBMC responses could be explained by a direct causal effect. The approach was to determine whether the *in vitro* addition of vitamin E to CBMC cultures altered proliferative and Th cytokine responses against a panel of mitogenic, antigenic and allergenic T-cell stimuli. The prime aim was to determine whether the *in vitro* addition of vitamin E to CBMC cultures at a physiological concentration observed in cord blood altered proliferative and Th-cell cytokine responses in a manner predicted by the original observation studies [12,13]. For comparison, we included control cultures supplemented with a second antioxidant, vitamin C, for which there is no evidence of any associations between maternal intake, maternal blood levels, CBMC responses and childhood wheeze/asthma [9,10,11].

Given the reported age related differential responsiveness of vitamin E on human Th-cell responses [4,8], a secondary aim of the study was to investigate the *in vitro* effects of vitamins E and C on adult peripheral blood mononuclear cell (PBMC) responses.

## 2. Experimental Section

### 2.1. Samples

Seventy mothers were recruited the day before scheduled elective caesarean section. After delivery, cord blood samples were collected to harvest serum, (in tubes with no anticoagulant) and CBMC, (in sodium heparin tubes). Blood was also obtained from adults, including 18 atopic patients attending the Chest Clinic at Aberdeen Royal Infirmary and 21 healthy volunteers recruited from University staff. This study was conducted according to the guidelines laid down in the Declaration of Helsinki and all procedures involving human subjects/patients were approved by the North of Scotland Research Ethics Committee. Written informed consent was obtained from all subjects/patients.

### 2.2. Cell Cultures

The cell culture methodology was identical to that used in our previous study that demonstrated the association between maternal vitamin E intake and CBMC responses [12]. As described previously, [12,16,17] CBMC and PBMC were isolated and cultured at a concentration of 1.25 × 10^6^ cells/mL in 2 mL wells, in Alpha Modification of Eagle’s Medium, with 2.5% complement inactivated autologous serum. CBMC and PBMC were stimulated with the following final culture concentrations of mitogen, antigens and allergens: Concanavalin A (con A) 2 μg/mL, *Mycobacterium tuberculosis* purified protein derivative 5 μg/mL (PPD), keyhole limpet haemocyanin (KLH) 5 μg/mL, house dust mite (*Dermatophagoides pteronyssinus*) extract (HDM) 1250 IU/mL (NIBSC, London, UK) or Timothy grass pollen extract (TG) 1250 IU/mL (NIBSC). Cell cultures were supplemented with either no antioxidant (controls), natural α-tocopherol (vitamin E), or ascorbic acid (vitamin C) (both Sigma, Gillingham, UK). Antioxidant supplementation was at final concentrations of 6.5 μmol/L for α-tocopherol and 44.11 μmol/L for ascorbic acid, corresponding to the cord blood 95th centile concentrations measured in the original general population study that reported an association between maternal vitamin E intake and CBMC responses [18]. Alpha-tocopherol was supplemented in 1% ethanol, appropriate control cultures demonstrated that at this concentration ethanol did not influence proliferative or cytokine responses.

### 2.3. Measurement of Responses

Assays of Th-cell responses have been described elsewhere [12,16,17]. Cell proliferation was assessed 5 days post-stimulation, by incorporation of ^3^H-thymidine in triplicate aliquots drawn from the cultures. Proliferative response was expressed as a stimulation index (SI), the ratio of mean counts/minute in stimulated *vs.* unstimulated control cultures [16,19,20]. Cellular enzyme linked immunosorbent assays (celELISA), [16] were carried out on day 5 after stimulation, [21] to measure the production of the Th1 cytokine interferon-γ (IFN-γ), the Th2 cytokine interleukin-4 (IL-4), and the regulatory cytokines IL-10 and transforming growth factor-β (TGF-β). Cytokine responses were also expressed as a SI, the ratio of cytokine production in stimulated *vs.* unstimulated control samples. Proliferative and cytokine responses were also expressed and analysed as absolute differences between stimulated and unstimulated responses.

### 2.4. Statistical Analyses

Based on our previous work [12], a study supplementing at least 40 cord blood samples had 80% power to detect a 20% difference in CBMC proliferation, 30% difference in IFN-γ secretion and a 15% difference in IL-4 secretion, at the 5% level of significance.

Our previous work [16] demonstrated that CBMC proliferative and cytokine responses approximate to log-normal distribution. In this study the proliferative responses approximated to log-normal distributions and the majority of cytokine responses to stimuli also approximated to log-normal distributions. Results were therefore presented as log-transformed data and initially analysed using paired *t* tests, with the level of significance taken as *p* < 0.05. A minority of cytokine responses (e.g., IL-4 and TGF-β responses to house dust mite and IFN-γ and IL-10 responses to timothy grass) did not approximate to log-normal distributions. A sensitivity analysis was therefore performed repeating the analysis using non-parametric tests (Wilcoxon) and these are the *p* values presented in the figures. In the preliminary analyses the PBMC responses from atopic and non-atopic adults did not differ significantly and there were no differential effects of supplementation, the combined responses of atopic and non-atopic PBMC donors are therefore presented. Analysis of proliferative and cytokine responses expressed as SIs and absolute differences were similar, the results of analysis of SI values are presented. Analyses were also stratified by high and low responses (SI cut point 3 for proliferation, 2 for cytokine responses), the results of this stratified analyses did not differ appreciably and are not presented.

## 3. Results

### 3.1. Vitamin E Supplementation of CBMC

**Figure 1:**
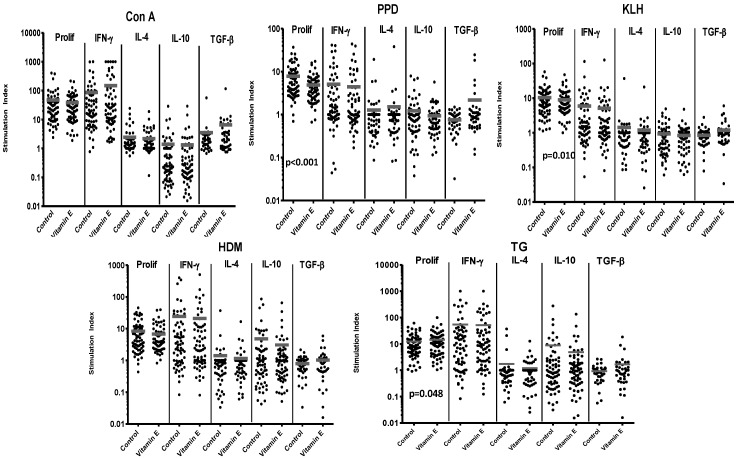
Responses of 70 cord blood mononuclear cell (CBMC) samples to control stimuli and allergens, with or without supplementation with vitamin E. Responses are expressed as stimulation index. Bars represent mean stimulation indices for each set of culture conditions. Significant *p* values for differences in responses between paired samples in cultures with or without vitamin E supplementation are shown on the relevant panels.

The addition of vitamin E to 70 CBMC cultures revealed complex effects. In general, this supplementation was associated with a trend for reduced proliferative responses to stimulation with the nominal antigens (KLH, PPD), or the allergen HDM (Figure 1).

The inhibition was significant for responses to PPD (57 of 67 cultures demonstrating a decrease) and KLH (36 of 66 cultures demonstrating a decrease). In contrast however, supplementation was associated with significantly increased proliferation in response to a second allergen, TG (43 of 69 subjects demonstrating an increase) (Figure 1 and Table 1-summary).

**Table 1:** Summary table representing the mean responses of CBMC and peripheral blood mononuclear cell (PBMC) samples to control stimuli and allergens, after supplementation with vitamin E or vitamin C. The mean change in response is shown for 70 CBMC samples and 18 PBMC samples, after supplementation with vitamin E, and for 51 CBMC and 18 PBMC samples, after supplementation with vitamin C. Significant *p* values for differences in responses between paired samples in cultures with or without vitamin E/vitamin C supplementation are shown on the relevant panels.

Vitamin E	Prolif	IFN-γ	IL-4	IL-10	TGF-β	Vitamin C	PROLIF	IFN-γ	IL-4	IL-10	TGF-β
*CORD*						*CORD*					
TG	↑ *p* < 0.05	↓ *p* < 0.05	↓	↓	↑	TG	↑	↑	↑ *p* < 0.05	↓	↓
HDM	↓	↓	↓	↓	↑	HDM	↓	↑	↑	↓ *p* < 0.05	↓
Con A	↓	↑	↓	↓	↑	Con A	↓ *p* < 0.05	↑	↑	↓	↓
PPD	↓ *p* < 0.001	↓	↑	↓	↑	PPD	↓	↑ *p* < 0.05	↑ *p* < 0.05	↓ *p* < 0.05	↓
KLH	↓ *p* < 0.001	↓	↓	↓	↑	KLH	↓	↑	↑ *p* < 0.05	↓	↓
*ADULT*						*ADULT*					
TG	↓	↑	↑	↑	↑	TG	↑ *p* < 0.05	↑	↑	↓	↑
HDM	↓	↑	↑	↑	↑ *p* < 0.05	HDM	↓	↑	↑	↓	↑
Con A	↓	↓	↑	↓	↑ *p* < 0.05	Con A	↑	↓ *p* < 0.05	↑	↓ *p* < 0.05	↑
PPD	↑	↓	↑	↓	↑	PPD	↓	↑	↑	↓	↑
KLH	↓	↓	↑	↑	↑	KLH	↓	↑	↑ *p* < 0.05	↓	↑

Overall, there was a trend for antigen or allergen CBMC cultures supplemented with vitamin E to exhibit decreased secretion of the cytokines IFN-γ, IL-10 and IL-4 (Figure 1 and Table 1), although production of TGF-β did not fit this pattern and was generally increased. Note that, even where significant effects were seen, there was considerable individual variation in the effects of vitamin E between different CBMC samples.

### 3.2. Vitamin E Supplementation of Adult PBMC

**Figure 2:**
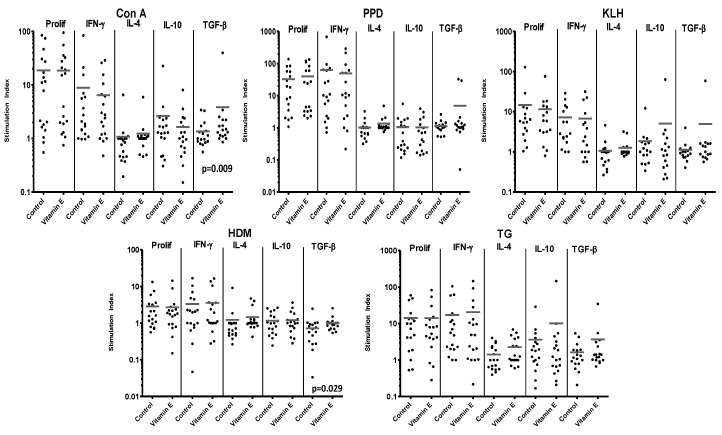
Responses of 18 PBMC samples to control stimuli and allergens, with or without supplementation with vitamin E. Reponses are expressed as stimulation index. Bars represent mean stimulation indices for each set of culture conditions. Significant *p* values for differences in responses between paired samples in cultures with or without vitamin E supplementation are shown on the relevant panels.

When the effects of vitamin E supplementation were tested on PBMC responses, unlike the results for CBMC, there were no consistent trends or significant differences in proliferation induced by the set of T-cell stimuli (Figure 2 and Table 1).

Likewise, there were no consistent trends in the effects of vitamin E supplementation on cytokine responses by PBMC. However, just as for CBMC, there was a trend for generally enhanced secretion of the regulatory cytokine TGF-β when PBMC were supplemented with vitamin E, and these increases were significant in the cultures responding to Con A (13 of 17 cultures exhibiting an increase) or HDM, (13 of 17 cultures exhibiting an increase) (Figure 2 and Table 1).

### 3.3. Vitamin C Supplementation of CBMC

**Figure 3:**
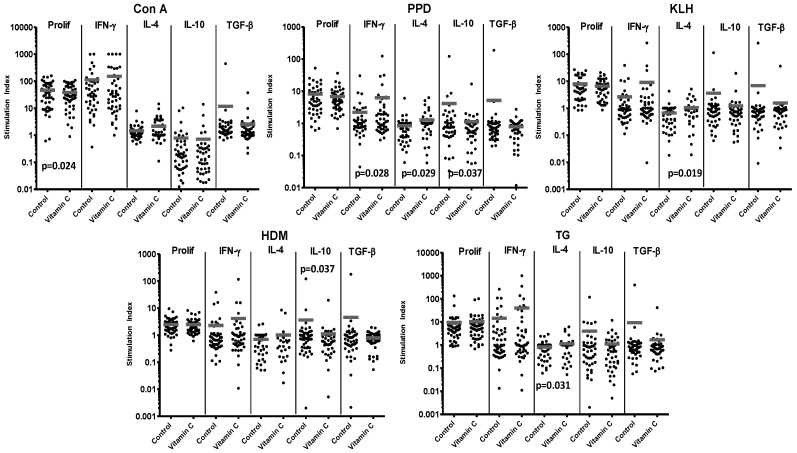
Responses of 51 CBMC samples to control stimuli and allergens, with or without supplementation with vitamin C. Reponses are expressed as stimulation index. Bars represent mean stimulation indices for each set of culture conditions. Significant *p* values for differences in responses between paired samples in cultures with or without vitamin C supplementation are shown on the relevant panels.

For comparison with the effects of vitamin E, 51 CBMC cultures responding to the set of T-cell stimuli were supplemented with vitamin C. There was no general effect of vitamin C on proliferation, although it did significantly reduce the proliferative response to Con A (30 of 48 cultures demonstrating a decrease) (Figure 3 and Table 1).

Unlike vitamin E supplementation, addition of vitamin C was associated with a general trend for increases in the effector cytokines IFN-γ and IL-4, which was significant for both cytokines after PPD stimulation (IFN-γ: 26 of 43 cultures demonstrating an increase in IFN-γ, IL-4: 20 of 42 cultures), and for IL-4 in cultures responding to KLH (21 of 41 cultures) or TG (20 of 46 cultures). However, vitamin C did reduce IL-10 responses, significantly in the case of PPD (23 of 48 cultures) and HDM (29 of 46) stimulation. The trend for enhanced TGF-β responses after vitamin E supplementation was not replicated for vitamin C, which generally reduced production of this regulatory cytokine, (Figure 3 and Table 1).

### 3.4. Vitamin C Supplementation on Adult PBMC

**Figure 4:**
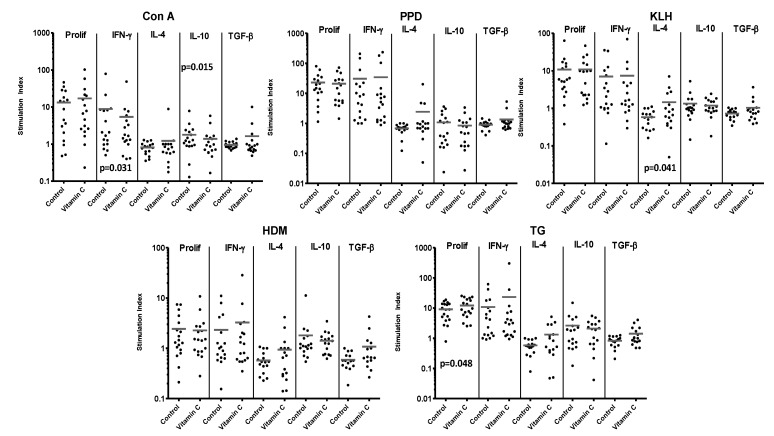
Responses of 18 PBMC samples to control stimuli and allergens, with or without supplementation with vitamin C. Reponses are expressed as stimulation index. Bars represent mean stimulation indices for each set of culture conditions. Significant *p* values for differences in responses between paired samples in cultures with or without vitamin C supplementation are shown on the relevant panels.

Vitamin C supplementation of PBMC was associated with trends for effects on proliferation that varied with different stimuli, but an increase in responses to TG was significant (12 of 18 cultures demonstrating an increase in proliferative responses) (Figure 4 and Table 1).

The effects of supplementation on the cytokine responses from PBMC cultures were also varied. However, as was the case for CBMC, in general, vitamin C supplementation was associated with a trend for an increase in cytokines characteristic of Th effector cells; IFN-γ and IL-4, which was significant for the IL-4 response to KLH, (10 of 16 cultures) although this trend was not universal and there was decreased IFN-γ after stimulation with Con A (Figure 4 and Table 1). Production of the regulatory cytokine IL-10 tended to be lower after vitamin C supplementation of PBMC, significantly so for Con A, whereas, unlike CBMC, there was a trend for TGF-β secretion to be increased.

### 3.5. Summary of Supplementation Results

**Figure 5:**
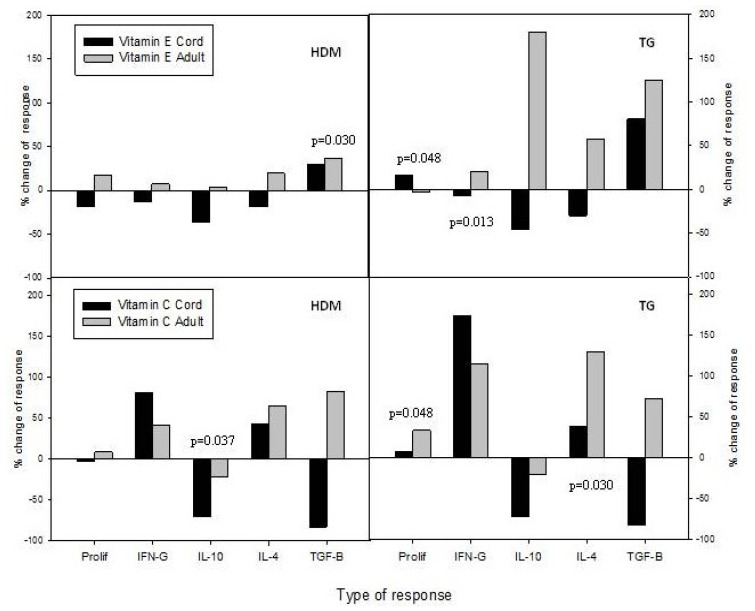
Summary of differential effects of vitamin E or C supplementation on CBMC or PBMC responses to allergen. Proliferation and secretion of IFN-γ, IL-10, IL-4 and TGF-β are expressed as the mean percentage change between paired unsupplemented and supplemented cultures. Significant *p* values for differences in responses between paired samples in cultures with or without vitamin supplementation are shown on the relevant panels.

The complex results of supplementation of CBMC and PBMC cultures with vitamins E and C are summarised for responses to the two allergens HDM and TG in Figure 5 and in Table 1.

However, despite the complexity, some patterns emerge. It can be seen that the effects of vitamin E supplementation on CBMC responses to these allergens were generally inhibitory, with the notable exceptions of stronger proliferation to TG and increased secretion of the regulatory cytokine TGF-β in cultures stimulated with either HDM or TG. In contrast, the pattern of effects of vitamin E supplementation on PBMC responses was typified by increases in both Th effector and regulatory cytokine responses. Vitamin C addition to cultures of either cell type also tended to enhance effector cytokine responses, whilst inhibiting IL-10 secretion, with TGF-β responses by CBMC also down-regulated.

## 4. Discussion

This study was based on our novel observation in a general population study, that low maternal vitamin E intake during pregnancy is independently associated with increased *in vitro* proliferative responses by CBMC and an increased likelihood of asthma in children aged 5 years. We were unable to demonstrate any associations between CBMC responses and maternal blood α-tocopherol at 10 weeks gestation and delivery, nor with cord blood α-tocopherol. Before embarking on a vitamin E intervention study during pregnancy we wanted to test whether *in vitro* vitamin E supplementation could modulate neonatal immune responses, reasoning that the demonstration of a direct effect would justify vitamin E supplementation, whereas failure to demonstrate a direct effect would justify alternative dietary strategies to increase vitamin E intake. Vitamin C was included as a control comparator given the absence of epidemiological evidence of an association between maternal vitamin C intake during pregnancy and CBMC responses and childhood asthma [9,10,11].

Previous work has reported low maternal vitamin E intake to be associated with increased CBMC proliferation to HDM and TG [13,16], encouraging the belief that the nutrient has a direct effect in modulating neonatal T-cell responses. This explanation predicts that that vitamin E supplementation of CBMC cultures would reduce proliferative responses. However, although direct supplementation of CBMC with vitamin E was associated with decreased proliferative responses to PPD, KLH and HDM, there was an opposite effect on proliferation to the allergen TG. This is an important differential result because, together with the between-subject variation and the complex effects on cytokine production, it indicates that the observed *in vivo* epidemiological association between maternal vitamin E intake during pregnancy and CBMC responses and childhood asthma/wheeze cannot be explained by a direct and consistent effect on overall Th-cell responsiveness, as originally hypothesised [22]. It is likely that the observed *in vivo* epidemiological associations with vitamin E intake reflect the effects of overall nutrient/dietary patterns associated with higher vitamin E intake. A further possibility raised by the current study is that isolated maternal vitamin E supplementation during pregnancy may have mixed or even adverse consequences for atopic sensitisation to different allergens. This concern is supported by the recent report that high-dose antenatal vitamin C and E supplementation is associated with increased infant healthcare utilisation and cost of care [23]. The differential effects of supplementation on HDM and TG responses excludes the possibility that any suppressive effects of vitamin E supplementation represent toxicity, which was also avoided by adding physiological concentrations representative of those measured in cord blood at birth in the epidemiological study demonstrating an association between vitamin E and CBMC responses [18].

It is unlikely that between subject variation in the neonatal serum added to the cultures contributed to the observed disparity between our epidemiological observations and the current study as the tocopherol present in 5% serum was negligible in comparison to the supplemental tocopherol. Differential associations between childhood fish intake and rye-grass and HDM sensitisation have been reported before and attributed to different timing of perennial and seasonal allergen exposure during early life [12,24]. This seems an unlikely explanation for the differential effects of vitamin E supplementation on TG and HDM responses seen in the current work because, as in our original observational study, CBMC samples were collected throughout the year [12].

The effects of vitamin E supplementation of CBMC cultures on cytokine responses were also not straightforward. On the one hand, the possible therapeutic use of vitamin E to help produce a “balanced” Th-cell profile in early life is supported by the finding that supplementation of CBMC with this nutrient generally inhibited cytokines characteristic of Th effector responses, including both Th1 (IFN-γ) and Th2 (IL-4) cytokines. On the other hand, supplementation of CBMC also had diverging effects on cytokines characteristic of Th regulatory responses, with trends for reduction in IL-10 levels but increases in TGF-β. Both IL-10 and TGF-β are immunosuppressive cytokines produced by a number of cell types, including the regulatory T cell subsets, and may therefore play a role in the prevention or control of allergic responses [25]. Particular regulatory subsets preferentially secrete either IL-10 or TGF-β, and so one explanation for the diverging effects of vitamin on the levels of these cytokines would be differential susceptibility of the respective subsets to supplementation. Vitamin E has been reported to influence regulatory T-cell function [26], which may in turn alter effector responses, but it is difficult to predict the overall consequences for immune function of the results of supplementation seen here.

This study followed on from our observation of an association between maternal vitamin E intake during pregnancy and CBMC proliferative responses [12]. Although the main focus of our work was the supplementation of CBMC, we included a parallel study of adult PBMC to examine the effect of the same *in vitro* vitamin E supplementation on adult PBMC in light of the reports of age related differential responsiveness. Vitamin E supplementation studies in colorectal cancer have reported a more pronounced stimulatory effect by vitamin E on naïve (CD45RA(+)) Th-cells as compared with T cells with a memory/activated phenotype (CD45RO(+)) [4]. Based on these reports we expected to observe differential effects of supplementation with CBMCs being more susceptible to the effects of supplementation than the adult PBMC. We found no convincing evidence of differential responsiveness of CBMC and adult PBMC to vitamin E supplementation; however the dose of vitamin E used in the current study was considerably less than that reported in previous work [4].

The current study had a number of limitations consequent upon the limited number of CBMC available from each sample. Only one dose of supplementation (equating to 95th centile concentrations in the cord blood) was used in this study, and so we cannot exclude a U-shaped dose response curve. However, the epidemiological data provide no evidence of a U-shaped dose response [9,10,11,12,13]. The single supplemental vitamin E dose was chosen because it would be representative, in a general population, of a clinically relevant vitamin E intervention resulting in a minimal twofold increase in cord blood α-tocopherol. However, supplementation *in vitro* does not load as efficiently as *in vivo* and so we are unable to assess the efficiency of the uptake into cells, which is a possible limitation. Nonetheless the tocopherol is clearly having an effect on the HDM and TG stimulated cells. Only single doses of mitogen and allergens were used to stimulate the CBMC; however these were identical to those used to identify the original association between maternal vitamin E intake and CBMC responses in a general population [12]. Furthermore, it should be noted that the responses seen here to allergens, may differ to that of other stimuli, such as LPS. Other stimuli were not included since our original observation was with allergens, and didn’t include LPS, furthermore there were a limited number of CBMC available from each sample. The population studied in the current study (children born by elective caesarean section) differed from the general population from which the original association between vitamin E and CBMC responses was drawn [12], however CBMC responses in a general population were not associated with mode of delivery (unpublished observations from [12]). The original association between maternal vitamin E and CBMC responses was independent of many potentially confounding factors (birth order, maternal atopy, maternal smoking, sex [12], and maternal BMI [27], making it unlikely that any effect of *in vitro* vitamin E supplementation was being negated by extraneous factors, moreover the effect of supplementation was compared with an unsupplemented control for each subject. A further limitation may arise given that we did not measure tocopherol or its isoforms in blood. However, our original observation was between maternal vitamin E intake and CBMC responses, we found no associations between CBMC responses and maternal (and cord) blood ascorbate tocopherol at 10 weeks gestation or at delivery [12].

A small number (*n* = 40) of adult PBMC samples were obtained to investigate a possible age related differential responsiveness to vitamin E. Consequently the concentrations of ascorbate and α-tocopherol used to supplement these PBMC cultures, was the same as that used to supplement CBMC cultures, however these levels are lower than those found in the plasma of healthy adults, which could explain the lack of effects seen in PBMC cultures. It should be noted however, that antioxidant supplementation studies in adults has not been shown to modify PBMC cytokine responses [28], which correlates with the results reported here. The proliferative data here were expressed using SI, as has been done in previous studies [19,20]. Although somewhat unusual to present cytokine data as SI’s, we also analyzed the proliferative and cytokine data using delta cpm (results not shown) and found that this made no difference to the observed associations or conclusions.

The mechanisms by which vitamins E and C mediated their complex effects on CBMC responses in the current study are unclear. It is possible these vitamins exert at least part of their influence through their antioxidant properties, since both provide important protection for maintaining cell membrane integrity, by limiting lipid peroxidation by reactive oxygen species [29]. However, such a shared property cannot provide an explanation where vitamins E and C differ in their effects on CBMC and PBMC responses. In these cases it is possible that the non-antioxidant properties of these vitamins have more potential to influence the first interaction between allergens and the immune system, and therefore may be responsible for many of the results seen in this study. Vitamin E is thought to modulate inflammatory processes by inhibiting the activity of nuclear factor kappa B (NFκB) [5], particularly in naïve, (CD45RA^high^) Th-cells, which may therefore be a mechanism by which it influenced the cord blood cells in the current study [30]. In the current study, the effects of *in vitro* vitamin E supplementation on CBMC and PBMC effector cytokine responses did differ, but the effects of *in vitro* vitamin C supplementation were broadly similar in neonates and adults. Whilst it is possible that this may reflect a differential response to supplementation by naive and antigen-experienced T-cells [4,8], it should be noted that a previous trial of *in vivo* antioxidant supplementation of atopic adults reported no association between changing parameters of serum antioxidant status and PBMC responses, again highlighting the disparity between *in vivo* and *in vitro* supplementation and the complexity underlying the reported associations between nutrient intake, asthma and atopic disease.

## 5. Conclusions

Overall, this work demonstrates that *in vitro* supplementation of CBMC with vitamins E or C can influence their immune function but not in the simple and consistent manner predicted by models arising from observational epidemiological studies. The results add to the body of work linking maternal diet to neonatal immune function and childhood asthma/wheeze [1,31,32], but highlight an important inconsistency between *in vitro* effects and *in vivo* associations. This disparity, in conjunction with recent antenatal intervention data [21] emphasises the complex nature of associations between nutrients and disease and the need carefully to consider the nature of any vitamin E based antenatal intervention intended to reduce the likelihood of childhood asthma. The findings of the current study have changed the direction of our vitamin E based intervention away from supplementation, towards dietary intervention, with vitamin E in its natural food based form [33].

## References

[1] Barker R.N., Burney P.G., Devereux G., Hardiman M., Holt P.G., O’Donnell M., Platts-Mills T.A., Seaton A., Strachan D.P., Weiss S.T. (2003). The increase in allergic disease: Environment and susceptibility. Proceedings of a Symposium held at the Royal Society of Edinburgh, 4th June 2002. Clin. Exp. Allergy.

[2] Seaton A., Godden D.J., Brown K. (1994). Increase in asthma: A more toxic environment or a more susceptible population?. Thorax.

[3] Devereux G., Seaton A. (2001). Why don’t we give chest patients dietary advice?. Thorax.

[4] Malmberg K., Lenkei R., Petersson M., Ohlum T., Ichihara F., Glimelius B., Frödin J.E., Masucci G., Kiessling R. (2002). A short-term dietary supplementation of high doses of vitamin E increases T helper 1 cytokine production in patients with advanced colorectal cancer. Clin. Cancer Res..

[5] Li-Weber M., Giaisi M., Treiber M.K., Krammer P.H. (2002). Vitamin E inhibits IL-4 gene expression in peripheral blood T cells. Eur. J. Immunol..

[6] Dunstan J.A., Breckler L., Hale J., Lehmann H., Franklin P., Lyons G., Ching S.Y., Mori T.A., Barden A., Prescott S.L. (2007). Supplementation with vitamins C, E, beta-carotene and selenium has no effect on anti-oxidant status and immune responses in allergic adults: A randomized controlled trial. Clin. Exp. Allergy.

[7] Yabuhara A., Macaubas C., Prescott S.L., Venaille T.J., Holt B.J., Habre W., Sly P.D., Holt P.G. (1997). TH2-polarized immunological memory to inhalant allergens in atopics is established during infancy and early childhood. Clin. Exp. Allergy.

[8] Meydani S.N., Han S.N., Wu D. (2005). Vitamin E and immune response in the aged: Molecular mechanisms and clinical implications. Immunol. Rev..

[9] Litonjua A.A., Rifas-Shiman S.L., Ly N.P., Tantisira K.G., Rich-Edwards J.W., Camargo C.A., Weiss S.T., Gillman M.W., Gold D.R. (2006). Maternal antioxidant intake in pregnancy and wheezing illnesses in children at 2 y of age. Am. J. Clin. Nutr..

[10] Miyake Y., Sasaki S., Tanaka K., Hirota Y. (2010). Consumption of vegetables, fruit, and antioxidants during pregnancy and wheeze and eczema in infants. Allergy.

[11] Devereux G., Turner S.W., Craig L.C., McNeill G., Martindale S., Harbour P.J., Helms P.J., Seaton A. (2006). Low maternal vitamin E intake during pregnancy is associated with asthma in 5-year-old children. Am. J. Respir. Crit. Care Med..

[12] Devereux G., Barker R.N., Seaton A. (2002). Antenatal determinants of neonatal immune responses to allergens. Clin. Exp. Allergy.

[13] Litonjua A.A., Tantisira K.G., Finn P.W., Schaub B., Schroeter C., Perkins D.L. (2004). Maternal antioxidant intake during pregnancy and cord blood lymphoproliferative responses. Am. J. Respir. Crit. Care Med..

[14] Weiss S.T., Litonjua A.A. (2007). Maternal diet *vs.* lack of exposure to sunlight as the cause of the epidemic of asthma, allergies and other autoimmune diseases. Thorax.

[15] Lawlor D.A., Davey Smith G., Kundu D., Bruckdorfer K.R., Ebrahim S. (2004). Those confounded vitamins: What can we learn from the differences between observational *vs.* randomised trial evidence?. Lancet.

[16] Devereux G., Hall A.M., Barker R.N. (2000). Measurement of T-helper cytokines secreted by cord blood mononuclear cells in response to allergens. J. Immunol. Methods.

[17] Marshall N.A., Vickers M.A., Barker R.N. (2003). Regulatory T cells secreting IL-10 dominate the immune response to EBV latent membrane protein 1. J. Immunol..

[18] Scaife A.R., McNeill G., Campbell D., Martindale S., Devereux G., Seaton A. (2006). Maternal intake of antioxidant vitamins in pregnancy in relation to maternal and fetal plasma levels at delivery. Br. J. Nutr..

[19] Miles E.A., Warner J.A., Jones A.C., Colwell B.M., Bryant T.N., Warner J.O. (1996). Periperhal blood mononuclear cell proliferative responses in the first year of life in babies born to allergic parents. Clin. Exp. Allergy.

[20] Jones A.C., Miles E.A., Warner J.O., Colwell B.M., Bryant T.N., Warner J.A. (1996). Fetal periperhal blood mononuclear cell proliferative responses to mitogenic and allergenic stimuli during gestation. Pediatr. Allergy Immunol..

[21] Beech J.T., Bainbridge T., Thompson S.J. (1997). Incorporation of cells into an ELISA system enhances antigen-driven lymphokine detection. J. Immunol. Methods.

[22] Devereux G. (2006). The increase in the prevalence of asthma and allergy: Food for thought. Nat. Rev. Immunol..

[23] Greenough A., Shaheen S.O., Shennan A., Seed P.T., Poston L. (2010). Respiratory outcomes in early childhood following antenatal vitamin C and E supplementation. Thorax.

[24] Andreasyan K., Ponsonby A., Dwyer T., Kemp A., Dear K., Cochrane J., Carmichael A. (2005). A differing pattern of association between dietary fish and allergen-specific subgroups of atopy. Allergy.

[25] Sakaguchi S., Miyara M., Costantino C.M., Hafler D.A. (2010). FOXP3+ regulatory T cells in the human immune system. Nat. Rev. Immunol..

[26] Tan P.H., Sagoo P., Chan C., Yates J.B., Campbell J., Beutelspacher S.C., Foxwell B.M.J., Lombardi G., George A.J.T. (2005). Inhibition of NF-kappa B and oxidative pathways in human dendritic cells by antioxidative vitamins generates regulatory T cells. J. Immunol..

[27] Devereux G., Seaton A., Barker R.N. (2002). Unpublished work.

[28] Dunstan J.A., Breckler L., Hale J., Lehmann H., Franklin P., Lyonso G., Ching S.Y.L., Mori T.A., Barden A., Prescott S.L. (2006). Associations between antioxidant status, markers of oxidative stress and immune responses in allergic adults. Clin. Exp. Allergy.

[29] Hughes D., Calder P.C., Field C.J., Gill H.S. (2002). Antioxidant Vitamins and Immune Function. Nutrition and Immune Function.

[30] Field C.J., Thomson C.A., van Aerde J.E., Parrott A., Euler A., Lien E., Clandinin M.T. (2000). Lower proportion of CD45R0+ cells and deficient interleukin-10 production by formula-fed infants, compared with human-fed, is corrected with supplementation of long-chain polyunsaturated fatty acids. J. Pediatr. Gastroenterol. Nutr..

[31] Dunstan J.A., Mori T.A., Barden A., Beilin L.J., Taylor A.L., Holt P.G., Prescott S.L. (2003). Fish oil supplementation in pregnancy modifies neonatal allergen-specific immune responses and clinical outcomes in infants at high risk of atopy: A randomized, controlled trial. J. Allergy Clin. Immunol..

[32] Chi A., Wildfire J., McLoughlin R., Wood R.A., Bloomberg G.R., Kattan M., Gergen P., Gold D.R., Witter F., Chen T. (2011). Umbilical cord plasma 25-hydroxyvitamin D concentration and immune function at birth: The Urban Environment and Childhood Asthma study. Clin. Exp. Allergy.

[33] Clark J., Craig L., McNeill G., Smith N., Norrie J., Devereux G. (2012). A novel dietary intervention to optimize vitamin E intake of pregnant women to 15 mg/day. J. Acad. Nutr. Diet..

